# Incorporating Neglected Insect Larvae in Species Inventories: DNA Barcoding as an Effective Tool for All‐Stage Invertebrate Identification in Tree Holes

**DOI:** 10.1002/ece3.71586

**Published:** 2025-06-24

**Authors:** Lucas Sire, Chloé Martin, Guilhem Parmain, Annie Bézier, Elisabeth A. Herniou, Christophe Bouget, Carlos Lopez‐Vaamonde

**Affiliations:** ^1^ Institut de Systématique, Évolution, Biodiversité (ISYEB), UMR7205 Muséum National d’Histoire Naturelle, CNRS Sorbonne Université, EPHE, Université des Antilles Paris France; ^2^ Institut de Recherche sur la Biologie de l’Insecte, UMR7261 CNRS Université de Tours Tours France; ^3^ INRAE EFNO ‘Forest Ecosystems’ Nogent‐sur‐Vernisson France; ^4^ INRAE, UR0633, Zoologie Forestière Orléans France

**Keywords:** Arthropoda, Berlese funnel trap, biodiversity, COI, tree‐related microhabitats, wood mold

## Abstract

Invertebrates, especially insects, are an integral part of biodiversity. Many species live in forest ecosystems where they play a key role in decomposing wood and maintaining ecosystem functions. Nevertheless, global changes, like fires, storms, and pest outbreaks, are impacting insect diversity, reinforcing the need for long‐term biomonitoring to understand and tackle these issues. Forests are heterogeneous ecosystems with tree‐related microhabitats (TReMs) such as tree holes, which are important for ecosystem diversity. Conventional identification approaches for species inventories are frequently hampered by the extensive and hidden diversity of insect larval stages. Thus, there is a crucial need to develop tools that facilitate inventories of these ecological niches and allow the incorporation of such hidden diversity into long‐term monitoring studies. To that end, we explored the biodiversity found in tree holes within French state forests using DNA barcoding and addressed challenges associated with traditional morphological identification methods. Results demonstrate the successful application of DNA barcoding in identifying nearly 62% of all invertebrates sampled from tree holes to the species level. Sampled invertebrates comprised 44% of larvae (566 individuals), of which nearly 50% could be assigned a species name. In total, 108 species and 173 barcode index numbers (BINs, used as species proxy) were molecularly inventoried, and 39% of these identified species were solely represented by larvae in our sampling. Our study highlights the usefulness of DNA‐based identification methods and the significance of including larvae in biodiversity assessments to gain insights into species abundance and functional diversity. It also underscores the necessity of ongoing and parallel developments of DNA reference libraries to improve species molecular identification rates and accuracy, and the need to investigate potential non‐destructive alternatives for biomonitoring. These efforts aim to ensure thorough and precise monitoring of invertebrate communities in tree holes and similar microhabitats.

## Graphical abstract

Insect inventories are often hampered by the hidden diversity of larval stages, but we demonstrate that DNA‐based identification methods are useful to gain insights into species diversity and abundance.

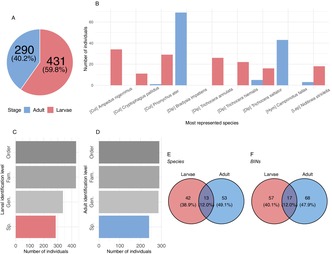

## 1 Introduction

Invertebrates, particularly insects, constitute the vast majority of life on Earth (Stork 2018), but their remarkable diversity and critical ecological roles remain largely undocumented. In forest ecosystems, one‐third of the entomofauna are saproxylic insects that help with wood decomposition and nutrient cycling (Ulyshen and Šobotník 2018). For example French forests host 2663 species of saproxylic Coleoptera (Bouget et al. 2019). However, insects have experienced significant declines in biomass (Hallmann et al. 2017) and changes in communities (Sire et al. 2022) across monitored areas. This is partly due to global changes that affect forests, such as increased fires, storms, and drought‐induced diebacks sometimes followed by pest outbreaks (Cours et al. 2022), but also directly linked to anthropogenic activities such as deforestation, agricultural intensification often associated with an increase in insecticide use (Wagner 2020). Therefore, it is important to establish long‐term monitoring programs that focus on invertebrates, especially insects, to track changes in biodiversity and identify essential forest features for invertebrate communities, such as tree‐related microhabitats (TReMs; Larrieu et al. 2018). This will enable the implementation of conservation and biodiversity‐friendly forest management programs (Sire et al. 2022).

Among forest habitats, tree‐related microhabitats (TreMs, Larrieu et al. 2018) are receiving increasing attention in forest ecology. One of the main forms of TreMs, that is, tree holes—cavities formed and enriched by decay and fungal activity—are uniquely stable microhabitats that support a succession of invertebrate and fungal communities throughout the wood decay process. They are created from heartwood, hence keeping the tree alive (Ranius et al. 2009), through dynamic processes such as mechanical damage or active excavations by cavity‐nesting vertebrates and/or the decomposition of rot holes by heart‐rot fungi in the center of trees (Jusino et al. 2015; Zawadzka et al. 2016). Over time, these holes become filled with soil‐like nutrient‐rich wood mold and water (Remm and Lõhmus 2011; Petermann and Gossner 2022). Tree holes serve various purposes, such as providing breeding sites, as well as food and shelter for a wide range of invertebrates, burrowing through the fungi‐infested dead wood and including keystone and endangered species (Gouix et al. 2015). Therefore, they are significant microhabitats that host a succession of invertebrates and fungi along the wood decay process, and it is crucial to survey and protect these reservoirs for forest biodiversity (Ranius et al. 2024). However, characterizing the invertebrate communities within tree holes poses major taxonomic challenges. Species richness is high, and many inhabitants are larval stages of holometabolous insects that lack clear morphological identification keys (Köhler et al. 2022; Gossner and Petermann 2022), not to mention the scarcity of expert taxonomists, which often limits biomonitoring, ecological, and functional studies to well‐known taxa disregarding the larval life stages. Insects also exhibit high temporal turnovers with changes in trophic functions throughout their life cycles (Gimmel and Ferro 2018). Consequently, the exclusion of hyper‐diverse, poorly known taxa or cryptic larval stages can introduce biases in abundance inferences and lead to an incomplete understanding of communities or misleading conclusions (Köhler et al. 2022; Domènech et al. 2022; Caterino and Recuero 2024). This taxonomic bottleneck in the identification of insect samples compounds the great impediment to understanding trends in forest biodiversity and to assessing fine‐scale functional and temporal turnover in invertebrate communities. It is particularly limiting in forest monitoring programs and critical for biodiversity conservation.

To address this taxonomic impediment, DNA‐based species delineation and identification, also known as DNA barcoding (Hebert et al. 2003) can be used to shed light on cryptic, unnamed, or unknown taxa, often referred to as “dark taxa” (Hartop et al. 2022; Chimeno et al. 2022). This approach involves comparing DNA barcodes with known references from curated and integrative databases such as BOLD (Ratnasingham and Hebert 2007). Importantly, it allows these dark taxa to be included as molecular operational taxonomic units (MOTUs) in biodiversity inventories and metabarcoding‐based studies (Cai et al. 2021; Sire et al. 2022; Chimeno et al. 2023). While near‐complete inventories of biodiversity in a particular environment or from passive‐trapping collections are becoming more accessible, the lack of Linnaean names and associated biological information limits the scope of holistic surveys and subsequent community analyses. This highlights the critical need for curated and comprehensive DNA reference libraries (Sire et al. 2019, 2022).

Here, we evaluate the utility of DNA barcoding as a relatively low‐cost and reliable tool for overcoming the taxonomic impediment by identifying insect larvae (Watanabe et al. 2024). We tackled this issue through a case study focusing on invertebrates, including insect larvae, collected from tree holes in two French forests. By integrating molecular identification with curated reference databases, we contribute new barcode data to expand DNA reference libraries for French forest invertebrates, thereby facilitating future monitoring and ecological studies. This study not only demonstrates the feasibility and advantages of applying DNA barcoding to both adult and larval communities in decaying tree cavities, but also provides a replicable approach for improving taxonomic resolution in biodiversity assessments of poorly known microhabitats. Our results highlight the value of molecular tools in revealing hidden diversity, especially in environments where conventional methods fall short.

## 2 Materials and Methods

### 2.1 Tree Holes and Specimen Samplings

We collected a total of 10 and 22 complete volumes of wood mold from tree holes in Rambouillet and Vierzon French state forests, respectively (Table S1). Hole‐bearing live trees were either pedunculate oak (*n* = 25; *Quercus robur* Linnaeus 1753; Rambouillet and Vierzon) or European beech (*n* = 7; *Fagus sylvatica* Linnaeus 1753; Vierzon only) and were sampled between January 27th and 30th of 2020 (Table S1). Each wood mold sample was collected with sterile spoons, placed separately in a plastic zip‐lock bags, and transported to the laboratory at room temperature. If samples were clogged, they were drained, and very large samples of wood mold were subdivided into smaller subsamples before being individually loaded into Berlese funnel traps. The Berlese funnel traps illuminated by a 150 W halogen lamp were active for 3 months (from February the 7th to May 14th, 2020), with weekly emptying, to recover the fleeing specimens in pure ethanol. Organisms retrieved were stored at −20°C awaiting individual sorting and tissue sampling.

Each individual was sorted, assigned to a unique sample‐ID, and photographed using a Leica DMS 300 (Leica Microsystems). A 2 min quick morphological inspection to identify each specimen to the finest rank possible (usually up to family level) involved checking major criteria (e.g., wings morphology, head sclerotization in larvae, etc.). It was then followed by a tissue sampling (i.e., ~1 mm of tissue at the abdomen level for larvae and one leg for imagines) individually placed in wells of 96‐well plates previously filled with 30 μL of pure ethanol. Tissue sampling was performed with forceps sterilized between each individual by cleaning with a 2% Decon 90 solution (Decon), rinsing with pure ethanol, and then heat sterilization at 300°C for 1 min in a glass‐microbeads sterilizer (Fisherbrand). All metadata (i.e., taxonomy, collecting data, etc.) and photographs of each individual were linked to their unique sample‐ID and uploaded to the Barcode of Life Data system (BOLD) (Ratnasingham and Hebert 2007) (https://boldsystems.org/). Remaining bodies of sampled organisms were kept individually with their given sample‐ID label in 2 mL screw‐capped tubes filled with pure ethanol and stored at 20°C. They are deposited at IRBI, Tours.

### 2.2 DNA Barcoding Laboratory Procedures and Analyses

All 96‐well plates filled with tissue samples were sent to the Canadian Centre for DNA Barcoding (CCDB) at the University of Guelph, Ontario, Canada for complete laboratory processing. DNA extraction was performed using the solid phase reversible immobilization (SPRI) method. PCR amplifications were done with primer cocktail C_LepFol (Hernández‐Triana et al. 2014) to target a 658 bp‐long fragment of the cytochrome *c* oxidase subunit I (COI) mitochondrial marker gene. PCR‐amplified samples were then sequenced using single molecule real‐time (SMRT) processing on PacBio Sequel sequencing platform (Pacific Biosciences, Menlo Parc—California, United States of America) (more information on standardized protocols for each step performed at the CCDB can be found here: https://ccdb.ca/resources/).

Sequence curation was done both automatically by the BOLD system and manually by individual BLAST*n* of consensus sequences to flag potential contaminations. Systematic errors in Sequel sequencing and associated false stop‐codon‐bearing sequences were corrected using Geneious *ver*. 11.1.5 (https://www.geneious.com/) (Kearse et al. 2012). Codon‐stop curation was performed by aligning the consensus sequence of the most similar and public BOLD hit with the MUSCLE alignment tool *ver*. 3.8.425 included in Geneious to check translation into protein to correct nucleotide errors from sequencing or to detect and discard potential NUMTs when necessary. Sequences were automatically assigned to barcode index numbers (BINs) by BOLD using the Refined Single Linkage (RESL) algorithm (Ratnasingham and Hebert 2013). The voucher taxonomy in BOLD was updated from BOLD‐ID engine recovery hits against the “species level barcode records” database, retaining species assignation for > 97% matches only and clarifying “Identification method” metadata category in BOLD. Additionally, reverse identification after BIN assignment was performed and corrected on the specimen page accordingly. All previous information on the morphologically‐assigned taxonomy was kept in the “Taxonomy notes” BOLD metadata for each newly curated voucher.

Statistical analyses were performed using R *ver*. 4.1.3 (R Core Team 2017). To compare species richness between life stage groups (i.e., larvae, adults and both included) and all invertebrates, normality of the data was assessed using Levene's test from the *car* package *ver*. 3.1‐0 (Fox and Weisberg 2019). Normality check of the residuals suggested the use of a non‐parametric Kruskal‐Wallis' test. Significance direction between tested groups was assessed by a pairwise Wilcoxon test with the *p*‐value adjusted using the Holm correction method. Community composition changes were tested using a glm test from the *mvabund* package *ver*. 4.2.1 (Cai et al. 2021) on the following model: community ~ cavity + stage + sector, family = binomial(“cloglog”), were “sector” stands for the two forests sampled. This analysis allows testing the importance of the listed parameters (i.e., cavities, life stage of collected insects and sample localization) in the species' depiction for each community, but also to explain variations in composition between them. Parameters' significance on community composition was then assessed using a type II Anova from the *car* package, to non‐sequentially and independently test parameters with no interaction effects. A total of 10 runs were computed, to which the post hoc Holm correction method was applied to validate significance statements. Ordination of the GLM model was done using the *ecoCopula* package *ver*. 1.0.2 (Popovic et al. 2019).

## 3 Results

### 3.1 DNA Reference Library Information on Invertebrates of Tree‐Holes

A total of 1343 invertebrate specimens were collected using Berlese funnel traps and sent for sequencing. After metadata and sequence curation, a total of 1287 records were retained for further analyses, of which 984 yielded good quality DNA barcodes (i.e., bearing < 1% *N* ambiguities). The sequencing success rate was 76.5% based on the curated dataset. The data are publicly accessible on BOLD under the dataset DS‐CAVITY at the following DOI: dx.doi.org/10.5883/DS‐CAVITY. Among the insect samples that did not successfully amplify, Coleoptera had the highest failure rate at 27%, with 82 failures out of 304 specimens analyzed, followed by Diptera at 9.9% and Hymenoptera at 9%. Notably, 120 out of the 303 vouchers that failed to generate a sequence could not be taxonomically classified into an order.

**FIGURE 1:**
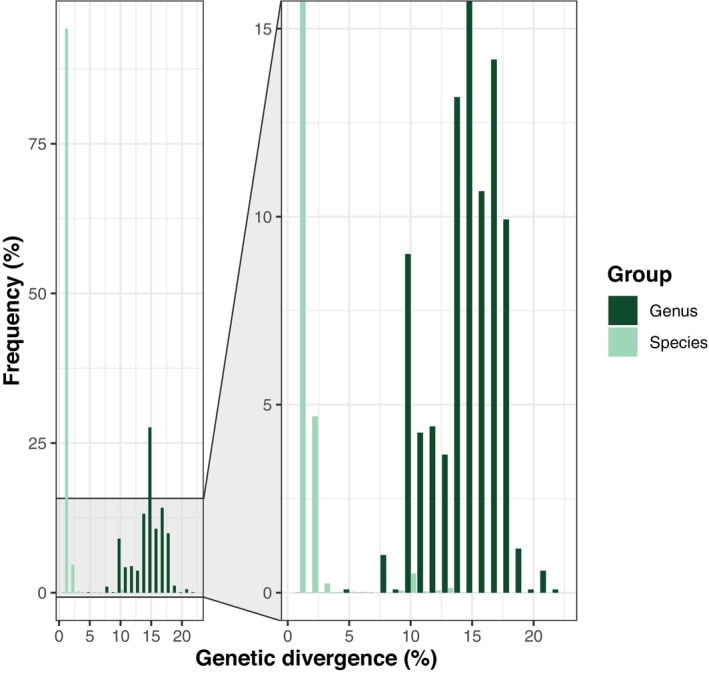
Barcode gap analysis of successfully sequenced specimens. Frequency of genetic divergence thresholds within species (light green) and between species (i.e., within genus—dark green) comparisons, respectively. Most of the genetic divergence within species groups (99% frequency) shows > 97% genetic similarity, while most of the within genus comparisons (96% frequency) are in a genetic divergence range of 10%–18% dissimilarity.

**FIGURE 2:**
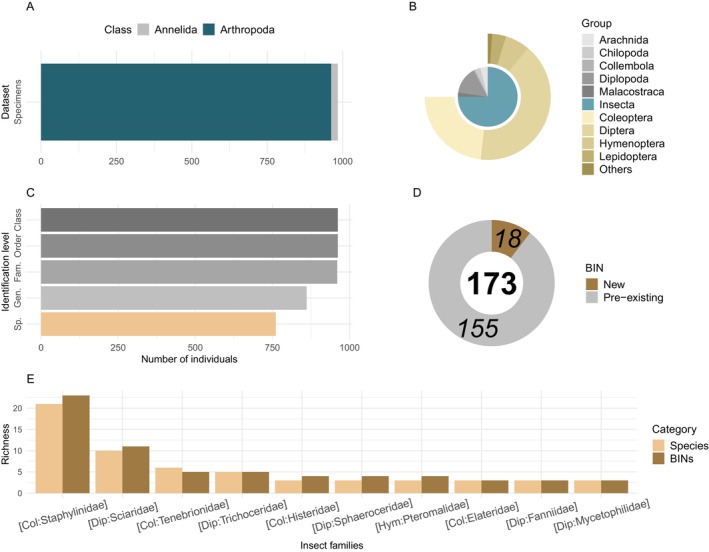
Taxonomic information on the invertebrate fauna recovered from tree holes and successfully sequenced. (A) Number of individuals of Arthropoda (blue) and Annelida (gray). (B) Taxonomic composition of individual arthropods based on DNA barcoding results compared to the BOLD system database, with the inner circle showing class level and outer circle order of insects recovered. “Others” category regroups Blattodea (5 individuals), Hemiptera (3), Neuroptera (1), Psocodea (1), Thysanoptera (1). (C) Total number of invertebrate specimens with taxonomic information from Class to Species level (from dark to light gray, with species level highlighted in light brown). Fam. = Family, Gen. = Genus, Sp. = Species. (D) Number of BINs that were already generated and available in BOLD (light gray) and newly added by this study (dark brown). The bold number indicates the total number of BINs found in the study. (E) Number of species (light brown) and BINs (dark brown) for the 10 richest Insect families found. Col = Coleoptera, Dip = Diptera, Hym = Hymenoptera.

The barcode gap was robust across the dataset, with 99% of intraspecific variability comparisons showing > 97% similarity, and 96% of interspecific variability falling within a range of dissimilarity ranging from 10% to 18% (Figure 1). In total, the 984 records accounted for 173 BINs with 18 new additions to BOLD (Figure 2D). Among the newly added BINs, four were identified as species, and seven were represented by insect larvae only. Integrating both morphology and molecular retro‐identification for these 173 BINs allowed for the successful identification of a total of 127 different species, seven of which are split cases (i.e., multiple BINs assigned to a single Linnean species name; Ratnasingham and Hebert 2013). To that extent, analyses focusing on Linnean species were performed using BINs with identical identification as merged, while those focusing on BINs with over‐splitting cases were kept separately. Identified species were mainly represented by Coleoptera (49) and Diptera (39). Overall, taxonomy could be assigned at the order level for all 984 barcoded records, for 982 (99.8%) at family, 883 (89.7%) at genus, and 783 (79.6%) at species level (Figure 2C; Table S2). Out of the successfully barcoded invertebrate specimens, 22 are annelids (2.2%; Figure 2A), the remainder being arthropods of which 721 are insects (73.3%; Figure 2B). The majority of insects are Diptera (392–54.4%), followed by Coleoptera (223–30.9%), the rest being Hymenoptera (61), Lepidoptera (34), Blattodea (5), Hemiptera (3), Neuroptera (1), Psocodea (1) and Thysanoptera (1) (Figure 2B). Overall, 60 insect families were represented, with Staphylinidae (Coleoptera) being the richest, both in terms of different species (21) and BINs (23) (Figure 2E).

### 3.2 DNA Barcodes of Larvae for Inventories and Monitoring

**FIGURE 3:**
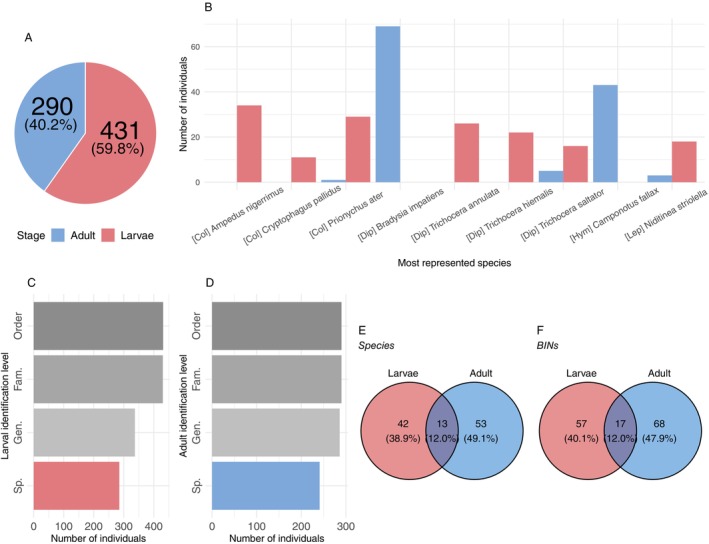
General information on the recovered insect biodiversity and their associated life stages. (A) Number of insect individuals of larvae (red) and imago (blue) successfully barcoded and the percentage it represents on the total number of insects sequenced. (B) Number of larval and adult specimens (in red and blue; respectively) for the 10 most abundant insect species found in the sampled tree holes. Col = Coleoptera, Dip = Diptera, Hym = Hymenoptera, Lep = Lepidoptera. (C, D) Total number of larval (C) and adult (D) individuals with identification level from Order to Species (from dark to light gray, with species level highlighted in red or blue), respectively. Fam. = Family, Gen. = Genus, Sp. = Species. (E, F) Venn diagram of the number of (E) identified species and (F) BINs exclusive to or shared between larval and adult life stages.

Insects at the larval stage accounted for 565 out of 950 (59.5%) of the total insect specimens recovered and 431 out of the 721 successfully sequenced insects (59.8%), resulting in a 76.3% sequencing success rate for larvae, similar to the 75.3% success rate for adults (Figure 3A). A total of 337 out of 431 sequenced larvae were identified to genus level (78.2%), with up to 285 larvae identified to species (66.1%) (Figure 3C). Of the 290 sequenced adults, up to 241 (83.1%) could be identified to species (Figure 3D). Among the nine most represented insect species in terms of sampled individuals, four were dipterans, three were coleopterans, and the remaining two were hymenopterans and lepidopterans (Figure 3B). Half of these species were represented by only one life stage—either larval or adult—while the other four showed the presence of both life stages in tree holes, but in varying proportions (Figure 3B). Upon closer examination of the 108 identified insect species, 42 species (38.9%) were represented solely by larval specimens, nearly half by adult specimens (53 species—49.1%), and only 13 species (12%) by both life stages (Figure 3E). Similarly, out of the 142 insect BINs in our dataset, 57 (40.1%) were composed of larval specimens only, 68 (47.9%) were represented by adult specimens only, and the remaining 17 BINs (12%) included both life stages (Figure 3F).

**FIGURE 4:**
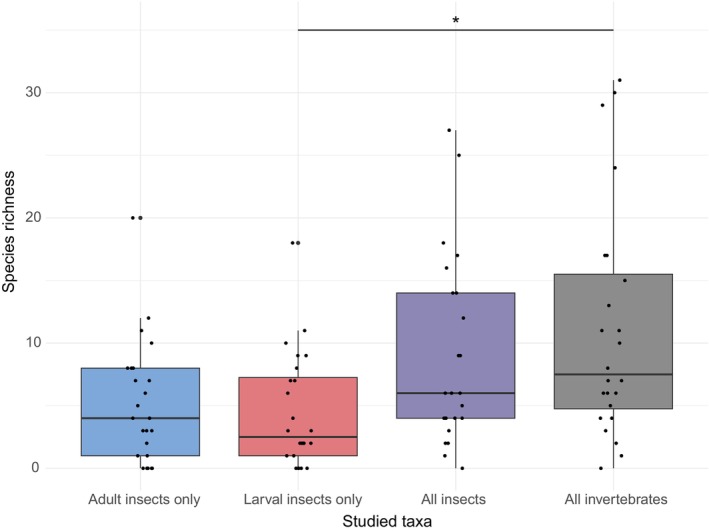
Diversity comparisons between taxonomic groups and developmental stages. Species richness per cavity was compared between taxonomic groups and developmental stages using BINs as species proxy. Insect life stage was assessed by comparing adults (blue) and larvae (red) individually, or combined (violet). All the invertebrate BINs sampled were also considered (dark gray). Statistical analyses highlighted a unique significant difference (annotated with “*”) in BIN richness between larvae and all the invertebrates (Wilcoxon pairwise test with Holm correction: Larvae—Invertebrates = 0.023).

**FIGURE 5:**
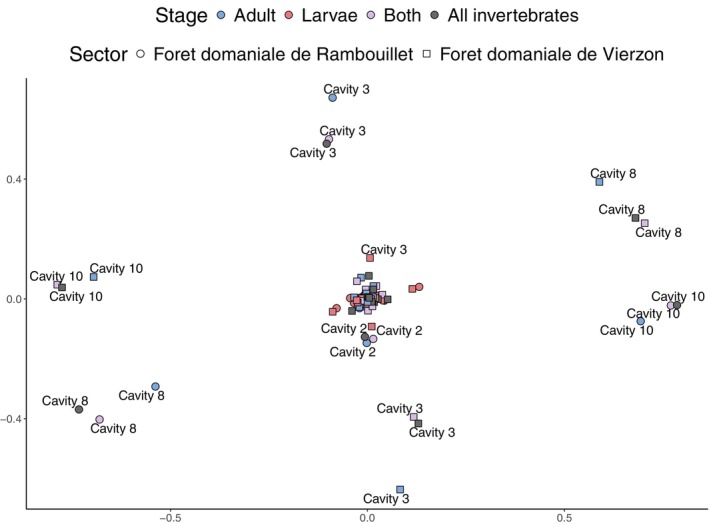
Variations in insect and invertebrate composition between sampled tree holes. Gaussian copula ordination was used to compare oinsect and invertebrate community compositions across cavities, accounting for geographic area (i.e., forest sampled), and for insects, life stages, using BINs as proxies for species. The importance of developmental stage was assessed by comparing community composition of insect adults (blue), larvae (red), both stages combined (violet), and all sampled invertebrates (dark gray). Mvabund analyses highlighted a significant impact of both the forest location and insect life stage on community composition dissimilarity between cavities, but no effect of the cavities themselves could be detected.

On average per cavity, insect BINs richness varied significantly between life stage groups of insects and the total invertebrate diversity found in tree holes (Kruskal‐Wallis rank sum test: *X* ^2^ = 12.442, df = 3, *p*‐value = 0.006012). However, pairwise comparisons adjusted with the Holm method showed no significant differences between insect developmental stages (*p‐*values: Larvae—Adult = 0.693; Larvae—Both = 0.079; Adult—Both = 0.211). When testing whether insect BINs richness for each life stage group could define the total species richness found in the cavities (i.e., including arthropods and other invertebrates), a significant difference was found only between the insect larval BINs diversity and the total invertebrate BINs richness (*p‐*values: Adult—Invertebrates = 0.062; Larvae—Invertebrates = 0.023; Both—Invertebrates = 0.693) (Figure 4; Table S3). Changes in BINs composition were nevertheless extremely significant depending on the life stage considered or between the two sampled forests (*p* < 0.001 after Holm correction method on 10 Anova runs), but not between tree holes (Figure 5).

## 4 Discussion

Incentives to monitor terrestrial insects are increasing, especially in forest stands. This is driven by the growing need to balance biodiversity with efficient forest management, aiming to mitigate more frequent and intense threats and avoid economic losses (Augustynczik et al. 2020). Unfortunately, large‐scale inventories based on morphology are hindered by the sheer richness of insects. This leads to initiatives focused on specific taxa, or to higher rates of misidentification by parataxonomists (Egli et al. 2020). These inventories are also limited by the three‐dimensional and heterogeneous structure of forest environments, which often restricts sampling to the easily accessible lower stratum. However, it is well‐known that canopies harbor unique and species‐rich diversity (Seifert et al. 2020). Taking these factors into account, this study emphasizes the usefulness of molecular tools, specifically DNA barcoding, in providing a more detailed inventory of diversity than traditional methods in other forest sub‐environments and tree‐related microhabitats of interest for biodiversity conservation, such as tree holes. Here, we confirmed the successful application of DNA barcoding in identifying species or delineating genetic species proxies, particularly when targeting hyperdiverse taxonomic groups such as invertebrates. Thus, we successfully sequenced over 75% (984 out of 1287 individuals) of all invertebrates found in the sampled tree holes, providing additional DNA barcodes for 173 BINs, including 18 new BINs to BOLD. These BINs span seven taxonomic Classes and 19 Orders.

Overall, our sequencing success was relatively high, regardless of the life stage (i.e., 76% for larvae, 75% for adults). This suggests that our tissue sampling from specimens collected in ethanol efficiently limited the addition of known biases to the wet‐lab and DNA amplification processes (e.g., variations in body size, incorporation of fatty acids, extra‐cuticular inhibitors). However, we found taxonomic discrepancies, with Coleoptera being in the order with the highest number of specimens that failed to produce sequences (82 out of 304 specimens, 27%). This low efficiency has been previously observed in collection specimens, where sequencing failures are often attributed to factors such as specimen age, trapping techniques, and preservation methods (Sire et al. 2019). Here, our study reveals that this relatively low amplification success also occurs with fresh specimens. Both studies used Folmer primers for DNA amplification in PCR, suggesting these primers may not be adequate for amplifying DNA from all coleopteran families (Hendrich et al. 2015). Therefore, there is a need for primers designed for specific taxonomic groups, or to develop them accordingly, to facilitate the production of DNA barcoding references and cover the wide diversity of coleopteran families during molecular inventory. While this would likely translate into a greater sequencing success, it would increase both the costs and the preparation time to sort and treat samples. Nevertheless, it remains particularly important because our second most represented sequenced taxon is Coleoptera with 223 individuals, more than a third of which (85 records) were identified as Staphylinids. Indeed, Staphylinidae is a species‐rich family and the most diverse in our study, with great ecological importance in forest ecosystem functioning (Bohac 1999). However, DNA reference libraries for this family are not relatively comprehensive, and there is a growing scarcity of taxonomic expertise (Krivosheeva et al. 2023).

DNA barcoding is a useful method for surveying tree holes and the associated wood mold because these environments often support a diverse range of larvae (Gossner and Petermann 2022). In Central Europe, a study focusing specifically on beetles associated with deadwood demonstrated the benefits of DNA barcoding by identifying to species nearly 93% of the 88% of larval specimens successfully sequenced (Köhler et al. 2022), thereby confirming the usefulness of European DNA reference databases for saproxylic beetles. While bearing a lower success likely attributable to the broader diversity of our sampling, our findings endorse the effectiveness of this approach for insects in general, as we were able to identify the species for 66% (285 individuals) of the sequenced larvae (431 individuals) that represented 60% of the total dataset of sequenced insects. In fine, DNA barcoding of larvae identified through reverse taxonomy may also help to build up a collection of reference specimens needed to develop determination keys for larval forms.

In our study, it is worth noting that the high proportion of larvae can be partly explained by our winter sampling, a period when many insects are in their overwintering stage and have not yet emerged as adults. Nevertheless, the inclusion of immature stages in inventories is important regardless of the season as it provides valuable information on a part of species abundance and diversity that is often overlooked (Domènech et al. 2022; Caterino and Recuero 2024). This was previously demonstrated by comparing larval and adult cohorts of saproxylic beetles, which were equally species‐rich but had significantly different species composition within tree holes (Köhler et al. 2022). Here, our results highlight a variability in species composition that is mainly driven by the adults, especially from some specific cavities. While we observed such variations in community composition between tree holes, it is important to consider that the micro‐environmental features of the tree holes were not assessed or controlled, and those few cavity outliers could greatly influence the observed differences, especially regarding the life stage comparisons. Nevertheless, larval stages indicate a developmental presence per se that requires the specificities of tree hole environmental features, potentially constraining its species diversity. In some cases, omitting larvae may thus not significantly affect species richness nor composition when multiple life stages coexist, but applies less to adults and their greater ability to disperse, allowing for a higher likelihood of visits and diversity of interactions (e.g., predation, avoidance behaviors, interspecific competition, or mating). This observation aligns with a community structuration following competition–colonization trade‐offs as described by Gossner and Petermann (2022). Still, including insect larvae can greatly enhance diversity inventories (Marcos‐García et al. 2012) and support ecological analyses of functional aspects, as different life stages may exhibit seasonal habitat shifts (Kirse et al. 2021) or distinct feeding behaviors with varying abundances in the sampled areas (Fikáček et al. 2024). Therefore, this information has implications for understanding the ecological processes at play in a given environment and can provide valuable insights into functional redundancies or specificities, which in turn can help inform appropriate conservation plans (Davis et al. 2023). On the practical aspects, alongside the reduced availability of taxonomist experts, the drop in sequencing costs (Hebert et al. 2025), together with an increased methodological advance in DNA‐based identification tools and associated DNA reference libraries, pushes toward an integrative taxonomy (Sire et al. 2019) that could improve identification of dark taxa and ease biodiversity monitoring overall (Köhler et al. 2022; Mrozińska and Obolewski 2023).

Although molecular‐based identification of invertebrates in tree holes has helped with species inventories, it is not without its flaws. Firstly, sampling costs (in terms of sequencing, additional collection efforts and techniques) need to be thoroughly compared with traditional monitoring approaches based on adult morphology. Secondly, the processes involved, such as manual or vacuum extraction (e.g., Bußler and Müller 2009), sieving, and Berlese methods, are invasive, leading both to the destructive sampling of the whole fauna and to the homogenization of the wood mold found in the tree holes. To mitigate the first aspect of destructive sampling, fieldwork testing of post‐sieving substrate replacement in tree holes—as well as the use of artificial cavities—have been shown to facilitate insect recolonization, even though the newly formed cohorts often differ significantly in richness and composition from the communities found in the natural tree holes (Petermann et al. 2016). The second aspect of homogenized substrate can negatively impact biomonitoring and conservation programs, as the recovered wood mold is only representative of a single sampling event and may have been disturbed and depleted. To partly address this issue, the use of sieved by‐products such as cuticular remains could provide a broader perspective on the historical diversity within the tree hole, similar to techniques used in archaeoentomology (Henríquez‐Valido et al. 2020), and thus allow reducing the sampling frequency needed to conduct long‐term biomonitoring programs. In a more promising way, eDNA methods on a small fraction of the substrate only could greatly reduce the homogenizing and destructive impacts of the current sampling, but further research needs to be conducted to determine whether they can serve as efficient and informative alternatives for biomonitoring tree holes.

## 5 Conclusion

DNA barcoding is a powerful tool for identifying invertebrates. This is particularly important when studying holometabolous insects and unique microhabitats such as tree holes. It enables us to include a wider range of species in comprehensive inventories or more detailed ecological and functional monitoring. However, there is still a need for continued development in DNA reference libraries to ensure accurate species identification. Additionally, further explorations into non‐destructive alternatives are necessary to expand our methods and knowledge.

## Author Contributions

**Lucas Sire:** conceptualization (supporting), data curation (equal), formal analysis (lead), methodology (lead), supervision (supporting), visualization (lead), writing – original draft (lead), writing – review and editing (equal). **Chloé Martin:** data curation (equal), formal analysis (supporting), writing – review and editing (equal). **Guilhem Parmain:** investigation (lead), writing – review and editing (equal). **Annie Bézier:** data curation (supporting), methodology (supporting), supervision (supporting), writing – review and editing (equal). **Elisabeth A. Herniou:** conceptualization (equal), supervision (supporting), writing – review and editing (equal). **Christophe Bouget:** conceptualization (equal), funding acquisition (equal), investigation (supporting), methodology (supporting), project administration (equal), writing – review and editing (equal). **Carlos Lopez‐Vaamonde:** conceptualization (equal), funding acquisition (equal), methodology (supporting), supervision (lead), writing – original draft (supporting), writing – review and editing (equal).

## Conflicts of Interest

The authors declare no conflicts of interest.

## Supporting information


**Table S1.** List of the sample locations.

**Table S2.** Synthetic table of the data recovered from the dataset DS‐CAVITY in BOLD database (dataset available at the following DOI: dx.doi.org/10.5883/DS‐CAVITY).

**Table S3.** Complementary information on sampling size and statistical results for the species richness comparisons between life stages.

## Data Availability

Data are publicly available at the following BOLD dataset: dx.doi.org/10.5883/DS‐CAVITY. Analytical script and processed data can be found at the following GitHub repository: https://github.com/Lucasire/CANOPE_larvae.git.
